# Neonatal invasive candidiasis in low- and middle-income countries: Data from the NeoOBS study

**DOI:** 10.1093/mmy/myad010

**Published:** 2023-03-06

**Authors:** Aislinn Cook, Laura Ferreras-Antolin, Bethou Adhisivam, Daynia Ballot, James A Berkley, Paola Bernaschi, Cristina G Carvalheiro, Napaporn Chaikittisuk, Yunsheng Chen, Vindana Chibabhai, Shweta Chitkara, Sara Chiurchiu, Elisavet Chorafa, Tran Minh Dien, Angela Dramowski, Samantha Faria de Matos, Jinxing Feng, Daniel Jarovsky, Ravinder Kaur, Warunee Khamjakkaew, Premsak Laoyookhong, Edwin Machanja, Marisa M Mussi-Pinhata, Flavia Namiiro, Gita Natraj, Hakka Naziat, Hoang Thi Bich Ngoc, Claude Ondongo-Ezhet, Kanchana Preedisripipat, Hafizur Rahman, Amy Riddell, Emmanuel Roilides, Neal Russell, Apurba S Sastry, Hannington Baluku Tasimwa, Ji Tongzhen, Jeannette Wadula, Yajuan Wang, Andrew Whitelaw, Dan Wu, Varsha Yadav, Gao Yang, Wolfgang Stohr, Julia Anna Bielicki, Sally Ellis, Adilia Warris, Paul T Heath, Michael Sharland

**Affiliations:** Centre for Neonatal and Paediatric Infection, St. George's University of London, London, UK; Centre for Neonatal and Paediatric Infection, St. George's University of London, London, UK; MRC Centre for Medical Mycology, University of Exeter, Exeter, UK; Department of Neonatology, Jawaharlal Institute of Postgraduate Medical Education & Research (JIPMER), Pondicherry, India; School of Clinical Medicine, Faculty of Health Sciences, University of Witwatersrand, Johannesburg, South Africa; Charlotte Maxeke Johannesburg Academic Hospital, Johannesburg, South Africa; Clinical Research Department, KEMRI-Wellcome Trust Research Programme, Kilifi, Kenya; Centre for Tropical Medicine & Global Health, Nuffield Department of Medicine, University of Oxford, Oxford, UK; The Childhood Acute Illness & Nutrition (CHAIN) Network, Nairobi, Kenya; Microbiology Unit, Bambino Gesù Children's Hospital, Rome, Italy; Department of Pediatrics, Ribeirão Preto Medical School, University of São Paulo, São Paulo, Brazil; Queen Sirikit National Institute of Child Health, Bangkok, Thailand; Clinical Laboratory, Shenzhen Children's Hospital, Shenzhen, China; Department of Clinical Microbiology & Infectious Diseases, School of Pathology, Faculty of Health Sciences, University of Witwatersrand, Johannesburg, South Africa; NHLS Microbiology Laboratory, Charlotte Maxeke Johannesburg Academic Hospital, Johannesburg, South Africa; Lady Hardinge Medical College & Associated SSK & KSC Hospitals, New Delhi, India; Academic Hospital Paediatric Department, Bambino Gesù Children's Hospital, Rome, Italy; Infectious Diseases Unit, 3rd Department of Pediatrics, School of Medicine, Faculty of Health Sciences, Aristotle University and Hippokration General Hospital, Thessaloniki, Greece; Vice Director Vietnam National Children's Hospital, Hanoi, Vietnam; Department of Surgery, Vietnam National Children's Hospital, Hanoi, Vietnam; Department of Paediatrics and Child Health, Faculty of Medicine and Health Sciences, Stellenbosch University, Cape Town, South Africa; Santa Casa de São Paulo, Sao Paulo, Brazil; Department of Neonatology, Shenzhen Children's Hospital, Shenzhen, China; Santa Casa de São Paulo, Sao Paulo, Brazil; Lady Hardinge Medical College & Associated SSK & KSC Hospitals, New Delhi, India; PHPT/IRD-MIVEGEC, Chiang Mai University, Chiang Rai, Thailand; Queen Sirikit National Institute of Child Health, Bangkok, Thailand; Department of Microbiology, KEMRI-Wellcome Trust Research Programme, Kilifi, Kenya; Department of Pediatrics, Ribeirão Preto Medical School, University of São Paulo, São Paulo, Brazil; Mulago Specialised Women and Neonatal Hospital, Kampala, Uganda; Seth G. S. Medical College & KEM Hospital, Mumbai, India; Child Health Research Foundation, Dhaka, Bangladesh; Department of Microbiology, Vietnam National Children's Hospital, Hanoi, Vietnam; School of Clinical Medicine, Faculty of Health Sciences, University of the Witwatersrand, Johannesburg, South Africa; Chiangrai Prachanukroh Hospital, Chiang Rai, Thailand; Child Health Research Foundation, Dhaka, Bangladesh; Centre for Neonatal and Paediatric Infection, St. George's University of London, London, UK; Infectious Diseases Unit, 3rd Department of Pediatrics, School of Medicine, Faculty of Health Sciences, Aristotle University and Hippokration General Hospital, Thessaloniki, Greece; Centre for Neonatal and Paediatric Infection, St. George's University of London, London, UK; Department of Microbiology, Jawaharlal Institute of Postgraduate Medical Education & Research (JIPMER), Pondicherry, India; Department of Mircobiology, College of Health Sciences, Makerere University, Kampala, Uganda; Beijing Obstetrics and Gynecology Hospital, Capital Medical University, Beijing, China; Beijing Maternal and Child Health Care Hospital, Beijing, China; National Health Laboratory Services, School of Pathology, Faculty of Health Sciences, University of the Witwatersrand, Johannesburg, South Africa; Department of Neonatology, Children's Hospital, Capital Institute of Pediatrics, 2# Yabao Road, Chaoyang District, Beijing, China; Department of Neonatology, Beijing Children's Hospital, National Center for Children's Health, Capital Medical University, Beijing, China; Division of Medical Microbiology, Faculty of Medicine and Health Sciences, Stellenbosch University, Cape Town, South Africa; National Health Laboratory Service, Tygerberg Hospital, Cape Town, South Africa; Department of Neonatology, Children's Hospital, Capital Institute of Pediatrics, 2# Yabao Road, Chaoyang District, Beijing, China; Seth G. S. Medical College & KEM Hospital, Mumbai, India; Beijing Obstetrics and Gynecology Hospital, Capital Medical University, Beijing, China; National Health Laboratory Services, School of Pathology, Faculty of Health Sciences, University of the Witwatersrand, Johannesburg, South Africa; MRC Clinical Trials Unit at UCL, Institute of Clinical Trials & Methodology, University College London, London, UK; Centre for Neonatal and Paediatric Infection, St. George's University of London, London, UK; Global Antibiotic Research & Development Partnership (GARDP), Geneva, Switzerland; MRC Centre for Medical Mycology, University of Exeter, Exeter, UK; Centre for Neonatal and Paediatric Infection, St. George's University of London, London, UK; Centre for Neonatal and Paediatric Infection, St. George's University of London, London, UK

**Keywords:** neonatal candidemia, low- and middle-income countries, *Candida parapsilosis*, candidiasis, *Candida auris*

## Abstract

Neonatal invasive candidiasis (NIC) has significant morbidity and mortality. Reports have shown a different profile of those neonates affected with NIC and of fluconazole-resistant *Candida* spp. isolates in low- and middle-income countries (LMICs) compared to high-income countries (HICs). We describe the epidemiology, *Candida* spp. distribution, treatment, and outcomes of neonates with NIC from LMICs enrolled in a global, prospective, longitudinal, observational cohort study (NeoOBS) of hospitalized infants <60 days postnatal age with sepsis (August 2018–February 2021). A total of 127 neonates from 14 hospitals in 8 countries with *Candida* spp. isolated from blood culture were included. Median gestational age of affected neonates was 30 weeks (IQR: 28–34), and median birth weight was 1270 gr (interquartile range [IQR]: 990–1692). Only a minority had high-risk criteria, such as being born <28 weeks, 19% (24/127), or birth weight <1000 gr, 27% (34/127). The most common *Candida* species were *C. albicans* (*n* = 45, 35%), *C. parapsilosis* (*n* = 38, 30%), and *Candida auris* (*n* = 18, 14%). The majority of *C. albicans* isolates were fluconazole susceptible, whereas 59% of *C. parapsilosis* isolates were fluconazole-resistant. Amphotericin B was the most common antifungal used [74% (78/105)], followed by fluconazole [22% (23/105)]. Death by day 28 post-enrollment was 22% (28/127). To our knowledge, this is the largest multi-country cohort of NIC in LMICs. Most of the neonates would not have been considered at high risk for NIC in HICs. A substantial proportion of isolates was resistant to first choice fluconazole. Understanding the burden of NIC in LMIC is essential to guide future research and treatment guidelines.

## Introduction

The World Health Organization (WHO) estimates that 2.4 million children died globally in the first month of life in 2019, with infection being the third commonest cause of death following prematurity- and intrapartum-related complications.^1^ The contribution of infection to deaths in the neonatal period is often underappreciated and varies according to geographic location, neonatal characteristics, and whether or not neonates are born in a medical facility.^2–4^

Neonatal invasive fungal infections are mostly caused by *Candida* spp. Reported rates of neonatal invasive candidiasis (NIC) vary significantly globally^5^ and are associated with a high crude mortality rate, ranging from 12% to 37% in high-income countries (HICs) and from 8.9% to 75% in low- and middle-income countries (LMICs).^6^ In HICs, NIC is most commonly reported in neonates <1000 gr birth weight or<28 weeks gestational age, but recent reports from LMIC neonatal units show the occurrence of NIC outside these specific groups.^3^,^7^,^8^ Although antifungal-resistant *Candida* spp. infections remain uncommon in HICs,^9^,^10^ LMICs are reporting an increasing proportion of fluconazole-resistant isolates, including *C. parapsilosis*,^11^,^12^*C. krusei*, and *C. auris*.^13^,^14^

The aim of this NeoOBS invasive candidiasis sub-study was to describe the epidemiology, antifungal resistance patterns, antifungal treatment, and clinical outcomes of neonates with *Candida* spp. bloodstream infections in LMICs. Data were collected as part of the larger NeoOBS study (https://clinicaltrials.gov/ct2/show/NCT03721302).

## Materials and methods

### NeoOBS study population

NeoOBS, a global, prospective, longitudinal, observational cohort study of hospitalized infants <60 days postnatal age with sepsis, was conducted at 19 hospitals in 11 countries, between August 2018 and February 2020. Hospitals were a mix of tertiary and district hospitals in Bangladesh (*n* = 1), Brazil (*n* = 2), China (*n* = 3), Greece (*n* = 1), India (*n* = 3), Italy (*n* = 1), Kenya (*n* = 1), South Africa (*n* = 3), Thailand (*n* = 2), Uganda (*n* = 1), and Vietnam (*n* = 1).^15^,^16^

**Figure 1.**
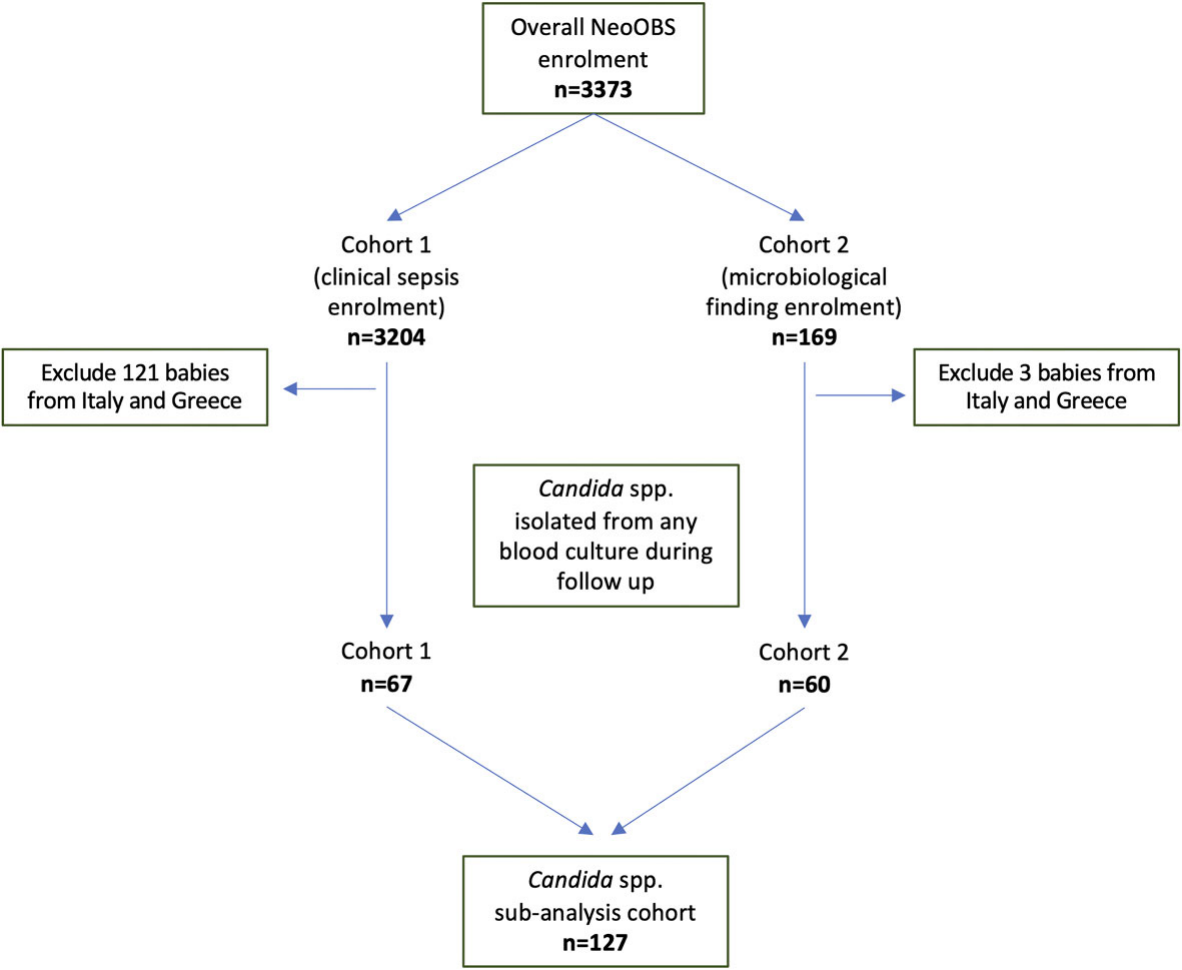
*Candida* spp. sub-study population derived from the overall NeoOBS study. See Supplementary Figure 1 for detailed schematic of the study population indicating the two enrollment cohorts. Note: Overall NeoOBS enrollment: cohort 1 was 3204 babies from 19 hospitals in 11 countries; cohort 2 was 169 babies from 14 hospitals in 10 countries. *Candida* sub-analysis cohort: cohort 1 includes 67 babies from 12 hospitals in 7 countries; cohort 2 was 60 babies from 12 hospitals in 7 countries.

Infants could be enrolled in the study in two different ways (Fig. 1). The primary cohort of infants was enrolled with clinical sepsis meeting the diagnostic criteria of at least one clinical sign of sepsis plus one clinical or laboratory sign, with a blood culture taken prior to initiating new antimicrobial treatment (referred to as cohort 1). Up to 200 infants from each hospital were enrolled through this route. Infants were excluded if the clinical signs were subsequently deemed to be more likely related to a non-sepsis diagnosis, as determined by the treating clinician (Supplementary Table 1).

In addition, a secondary cohort of infants (referred to as cohort 2) was enrolled based on the isolation of a carbapenem-resistant organism or *Candida* spp. from blood culture or with confirmed bacterial meningitis (Supplementary Table 1). Cohort 2 was designed to better understand specific infections and capture infants with these infections who may not have been enrolled in or eligible for cohort 1. There was no minimum or maximum enrollment number for cohort 2 across hospitals. Infants already enrolled in cohort 1 were not eligible to be enrolled in cohort 2 as all microbiology findings were already captured as part of cohort 1 follow-up.

Exclusion criteria for both cohorts were significant non-infectious-related comorbidity expected to cause death within 72 h, enrollment in an interventional study or previous enrollment in this study. Hospitals were given pragmatic flexibility for enrollment time frames given variability in case numbers and staffing capacity. Full inclusion and exclusion criteria for both cohorts are described in Supplementary Table 1.

### Data collection

Infants meeting the eligibility criteria were enrolled in both cohorts. Infants in cohort 1 were followed prospectively daily for the duration of hospitalization up to day 28 from the day of enrollment.

For infants in cohort 2, daily clinical and antimicrobial treatment data and any laboratory or microbiological investigations were retrospectively collected using medical notes and other available data from the day the culture was taken up to the day of enrollment, and then, prospectively collected from the day of enrollment (Supplementary Fig. 1) to 28 days from when the eligible blood culture was taken. For babies in both cohorts, clinical signs, supportive measures, and antimicrobial treatment were collected daily from the day of enrollment; blood culture, routine laboratory investigations, and other microbiology results were collected as and when conducted. At enrollment, demographics, labor and delivery details, and risk factors were collected.

All treatments and investigations were at the discretion of clinicians at the local hospital and were not determined by the study processes. At discharge or in-hospital death, information on mortality (if applicable), antimicrobial treatment, and both infection- and non-infection-related diagnoses were collected. Infants who were discharged prior to day 28 were telephoned on day 28, to assess vital status and any medical interventions since discharge. The primary outcome of the study was death by day 28, from the day the enrollment blood culture was taken. Primary and secondary causes (if applicable) of death were captured both for infants who died in hospital and those who died post-discharge before day 28.

Microbiological examinations were conducted as per local hospital procedures; however, babies must have had a blood culture taken prior to new antimicrobials being started to be eligible for enrollment in cohort 1. Blood culture results were collected as reported by local hospitals.

Study data were collected by paper case report form and entered and managed using REDCap electronic data capture tools hosted at St. George's, University of London. REDCap is a secure, web-based software platform^17^,^18^ used for the collection and management of research data.

Ethics approval from local and national bodies was received by each hospital prior to commencing recruitment. Informed consent was obtained for all patients prior to enrollment.

### Candidemia study population

All infants enrolled in the NeoOBS study via cohort 1 or cohort 2 who had *Candida* spp. isolated from a blood culture at any point during their follow-up up to day 28 (regardless of enrollment diagnosis) were included in this analysis. Analyses were restricted to the first *Candida* spp. isolated from each patient. Regardless of when during follow-up the *Candida* spp. was taken, all infants were censored at 28 days from when the enrollment blood culture for the overall NeoOBS study was taken (see Supplementary Fig. 1) This means some infants may have contributed fewer than 28 days of follow-up from when first *Candida* spp. culture was taken to this candidemia sub-analysis.

Due to differing times in follow-up when *Candida* spp. cultures were taken for these patients, for this sub-analysis, all patients were aligned with day 0 defined as the day the positive *Candida* spp. blood culture was taken (Supplementary Fig. 1). All data collection tools were the same for infants in both cohorts. Analyses were restricted to infants from LMICs only, thus excluding infants from Greece (*n* = 3) and Italy (*n* = 0).

### Statistical analysis

Categorical variables were described as relative frequency, and continuous variables were described as median and interquartile range (IQR). Demographic and clinical characteristics between enrollment cohorts were compared using the *χ*^2^ test. Kaplan–Meier curves and Cox proportional hazards model were used to investigate mortality. All data management and analyses were conducted in RStudio v1.4.1717 (R version 4.0.3).

## Results

### Study population and baseline characteristics

After excluding infants from Greece and Italy, 3083 neonates were enrolled from 17 hospitals in cohort 1, and 166 neonates were enrolled from 14 hospitals in cohort 2. Results from the overall NeoOBS study are described elsewhere.^19^ Overall, 127/3249 (4%) infants met the inclusion criteria for the candidemia sub-analysis (67 were from cohort 1 and 60 from cohort 2) (Fig. 1). Infants with candidemia were from 14 hospitals in eight LMICs; however, 85% (108/127) of the infants with candidemia were reported from seven hospitals in three countries (South Africa, India, and Vietnam). Forty-six percent (58/127) of infants had *Candida* spp. isolated during follow-up after enrollment in the overall NeoOBS study, while the remaining 54% (69/127) had *Candida* spp. isolated from their baseline enrollment blood culture.

At the time when the blood culture that grew *Candida* spp. was taken, the median postnatal age was 16 days (IQR: 10.5–21), the median gestational age at birth was 30 weeks (IQR: 28–34), and the median birth weight was 1270 gr (IQR: 990–1692). Fifty-four percent (68/127) of the infants were male. Only 19% (24/127) of the infants were born before 28 weeks of gestation, and 27% (34/127) had birth weights <1000 gr. Infants with candidemia were hospitalized for a median of 14 days (IQR: 6.5–20) prior to when the *Candida* spp. blood culture was taken. Eighty percent (102/127) of infants received at least one broad-spectrum antibiotic in the week prior to that blood culture. The majority of cases, 90% (114/127), were born either at the enrolling hospital or in a referral hospital and remained hospitalized from birth. Baseline characteristics of both enrollment cohorts were similar (Supplementary Table 2). Epidemiological characteristics by survival are summarized in Table 1.

**Table 1.** Comparison of summary characteristics of neonates with candidemia by survival status.

	Overall (*n* = 127)	Survived (*n* = 99)	Died (*n* = 28)
Sex; Female (%)	59 (47)	44 (44)	15 (54)
Birth weight (gr) (median [IQR])	1270.0 [990.0, 1692.5]	1300.00 [1022.5, 1724.5]	955.00 [772.0, 1655.0]
Gestational age (weeks) (median [IQR])	30 [28, 34]	30 [28, 34]	29 [27, 33]
Age at *Candida* spp. culture (days) (median [IQR])	16 [10.5, 22.0]	16.0 [12.0, 22.0]	14.5 [8.0, 22.5]
Birth status Hospitalized since birth (%)	114 (90)	90 (91)	24 (86)
Organism (*n* = 128) (%)			
*Candida albicans*	45 (35)	35 (35)	10 (36)
*Candida parapsilosis*	38 (30)	31 (31)	7 (25)
*Candida auris*	18 (14)	13 (13)	5 (18)
Other *Candida* spp.^a^	27 (21)	21 (21)	6 (21)
Country (%)			
India	40 (32)	30 (30)	10 (36)
South Africa	55 (43)	44 (44)	11 (39)
Vietnam	13 (10)	10 (10)	3 (11)
Other^b^	19 (15)	15 (15)	4 (14)
Hospital (%)			
Hospital 1	28 (22)	22 (22)	6 (21)
Hospital 2	25 (20)	20 (20)	5 (18)
Hospital 3	21 (17)	17 (17)	4 (14)
Hospital 4	13 (10)	10 (10)	3 (11)
Hospital 5	11 (9)	8 (8)	3 (11)
Other^c^	29 (23)	22 (22)	7 (25)

^a^Other *Candida* spp. include *C. famata* (*n* = 1), *C. glabrata* (*n* = 6), *C. metapsilosis* (*n* = 1), *C. pelliculosa* (*n* = 4), *C. rugosa* (*n* = 1), undefined *Candida* spp. (*n* = 10), and *C. tropicalis* (*n* = 4).

^b^Other countries are comprised of five countries, each contributing <8 participants (range: 1–7 per country).

^c^Other sites are comprised of nine hospitals, each contributing <7 participants (range: 1–6 per site).

### Microbiology findings

The most common *Candida* species in this study were *C. albicans* (*n* = 45, 35%), *C. parapsilosis* (*n* = 38, 30%), and *C. auris* (*n* = 18, 14%). Other species isolated were *C. glabrata* (*n* = 6*), C. pelliculosa* (*n* = 4)*, C. tropicalis* (*n* = 4)*, C. famata* (*n* = 1)*, C. metapsilosis* (*n* = 1), and *C. rugosa* (*n* = 1). There were 10 (8%) unspecified *Candida* spp. (Table 1). Species distribution varied by hospital (*P *< .0001) and country (*P *< .0001) (Supplementary Table 3). One patient had two *Candida* spp. isolates (*C. parapsilosis* and *C. glabrata*) from the same blood culture (a total of 128 *Candida* spp. isolates). Sixty-one percent (11/18) of *C. auris* isolates were found in India, and the remaining 39% (7/18) were from South Africa.

**Figure 2.**
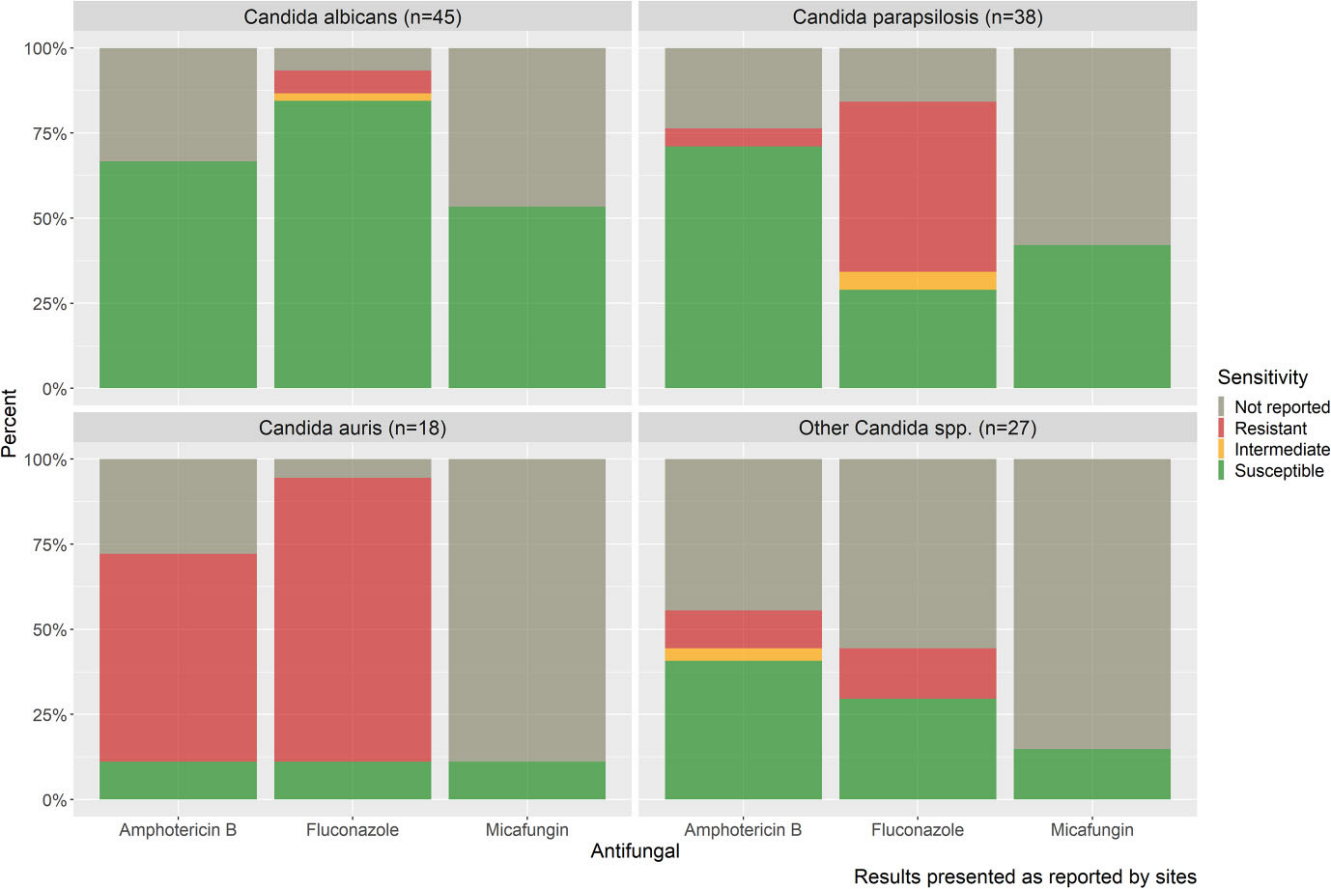
Reported susceptibility profiles to amphotericin B, fluconazole, and micafungin for the most common *Candida* species.

Susceptibility testing was not reported for 13% (16/128) of isolates, and for 17 isolates (13%), only fluconazole susceptibility was reported. Susceptibility results for fluconazole, amphotericin B, and an echinocandin [micafungin] were reported in 80% (103/128), 78% (87/128), and 36% (46/128) of all *Candida* spp. isolates, respectively (Fig. 2). Of these, overall, 41/103 (40%) were fluconazole-resistant, 16/87 (18%) were amphotericin B-resistant, and 0/46 (0%) were micafungin-resistant.

Of the *C. albicans* isolates with susceptibility results reported, 91% (38/42) were susceptible to fluconazole, and 100% (30/30) were susceptible to amphotericin B; however, 15/45 (33%) did not have amphotericin B susceptibility reported.

Reported resistance to fluconazole in *C. parapsilosis* was high (19/32 resistant, 59%); however, the majority were susceptible to amphotericin B (27/29 susceptible, 93%). Almost all the fluconazole-resistant *C. parapsilosis* isolates (17/19, 90%) were reported from South Africa.

There was an expected high reported resistance to fluconazole (15/17, 88%) and amphotericin B (11/13, 85%) in *C. auris* isolates. Most *C. auris* isolates did not have micafungin susceptibility testing done (16/18, 89%). Resistance of *C. auris* isolates to voriconazole was reported in 31% (5/16). Figure 2 illustrates the susceptibility profiles of *Candida* spp. isolates to the three commonly used antifungal agents.

### Antifungal drug use

Overall, 14% (18/127) of infants received antifungals for either prophylaxis (*n* = 8) or empirical treatment (*n* = 11) in the week preceding the positive blood culture with *Candida* spp. being taken. Of those, all the infants who received prophylaxis received fluconazole. Of those receiving empirical treatment prior to the blood culture, amphotericin B was received by 73% (8/11) of infants.

Overall, neonatal antifungal prophylaxis was uncommon in the infants that developed candidemia (8/127, 6%). None (0/27) of those born before 28 weeks of gestation, and only 3/34 (9%) of neonates with a birth weight <1000 gr received prophylaxis.

Ninety percent of infants (114/127) received antifungal treatment after taking the blood culture that grew *Candida* spp., including eight infants who continued antifungal treatment that had been started before the culture (fluconazole: *n* = 3 and amphotericin B: *n* = 5). Of the 106, who started any new antifungals after the blood culture was taken, only one had received fluconazole prophylaxis. After taking the blood culture, the median time to start a new antifungal treatment in these 105 infants was 3 days (IQR: 2–4). The first antifungal treatment of choice was amphotericin B in 74% (78/105) of the cases and fluconazole in 22% (23/105) of the cases.

Out of 88 infants, 85 (97%) received appropriate antifungal treatment based on the reported *in vitro* susceptibility profile of the *Candida* species with available susceptibility testing results. In these infants, amphotericin B (*n* = 45) was the most commonly prescribed antifungal, followed by fluconazole (*n* = 27), voriconazole (*n* = 10), and micafungin (*n* = 3). In infants with known susceptibility profile of the *Candida* species, the median time to appropriate antifungal treatment was 3 days from when the blood culture was taken (IQR: 2–5 days, range: 8 days prior to blood culture to 12 days after blood culture).

**Figure 3.**
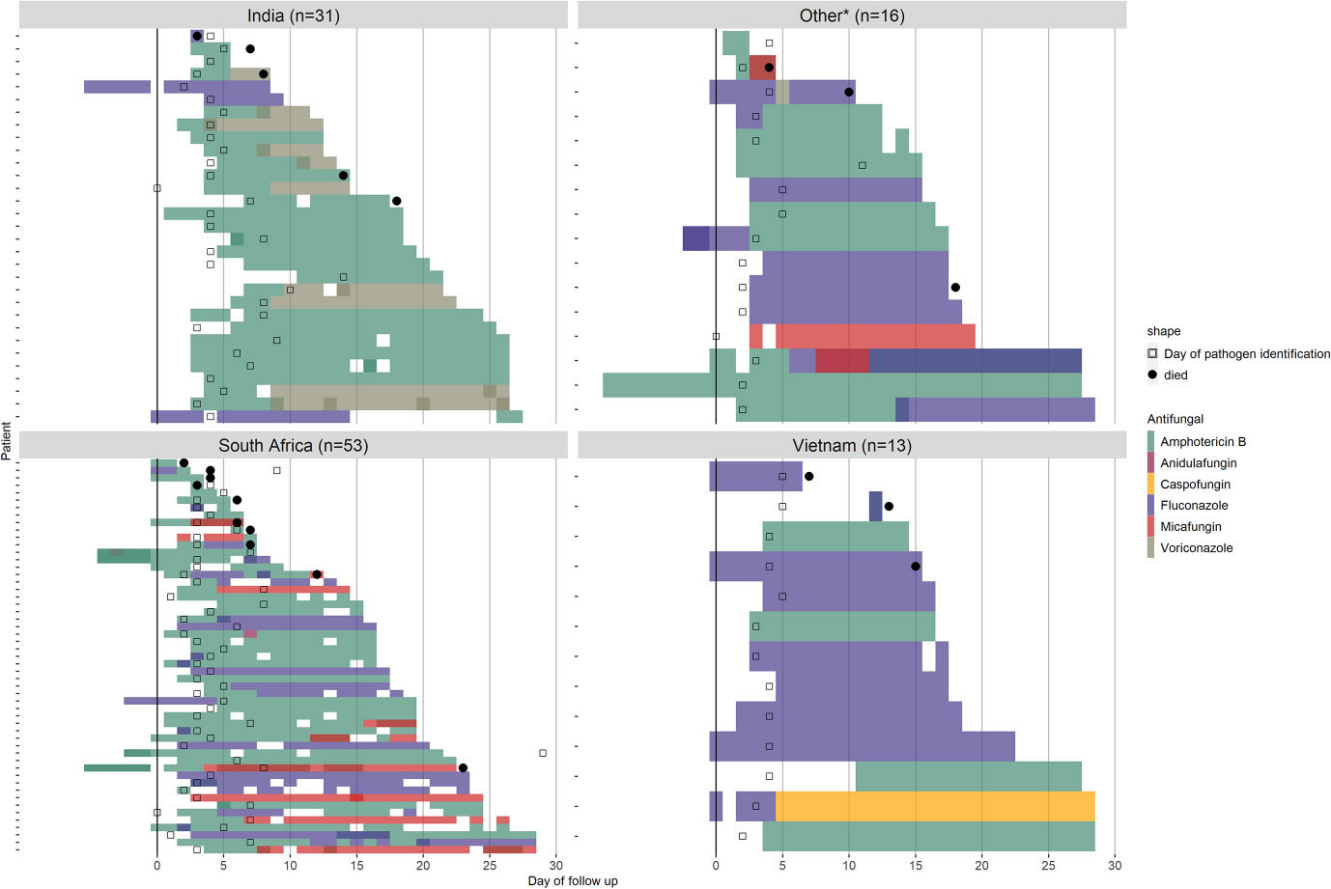
Patient-based antifungal treatment choice by country indicating the day the blood culture was taken (day 0), the day the fungal organism was identified (open squares), and mortality (solid dots). White space indicates calendar days that antifungal treatment was not given. *Other countries are comprised of five countries, each contributing < 8 participants (range: 1–7 per country).

**Figure 4.**
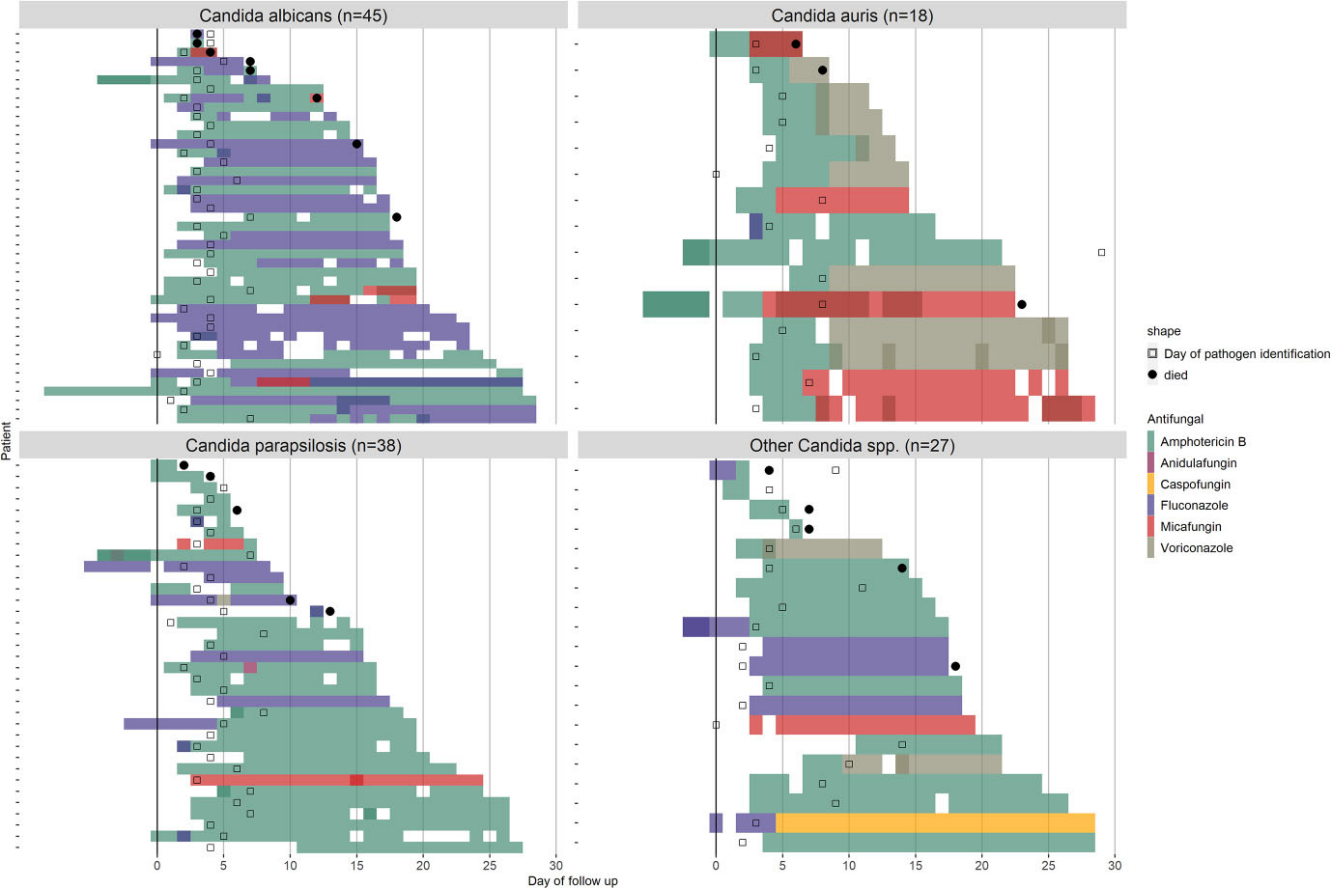
Patient-based antifungal treatment choice by causative *Candida* spp. Day 0 is the day the blood culture was taken that grew *Candida* spp. Day that the *Candida* spp. was identified is indicated with open squares and mortality with solid dots.

Antifungal treatment varied by country (Fig. 3) and causative *Candida* species (Fig. 4). Amphotericin B was used in all countries. In infants who received antifungal treatment after blood culture, a higher proportion of infants received amphotericin B in South Africa (48/53, 91%) and India (28/31, 90%) compared to Vietnam (5/13, 38%) (Fig. 3). Fluconazole was less commonly prescribed in India (4/31, 13%) and South Africa (23/53, 43%) compared to Vietnam (9/13, 69%). Voriconazole was used only in India (*n* = 10), micafungin was used predominantly in South Africa (*n* = 10), and caspofungin was used only in Vietnam (*n* = 1). Infants may have received more than one antifungal during their treatment.

### Clinical outcome

**Figure 5.**
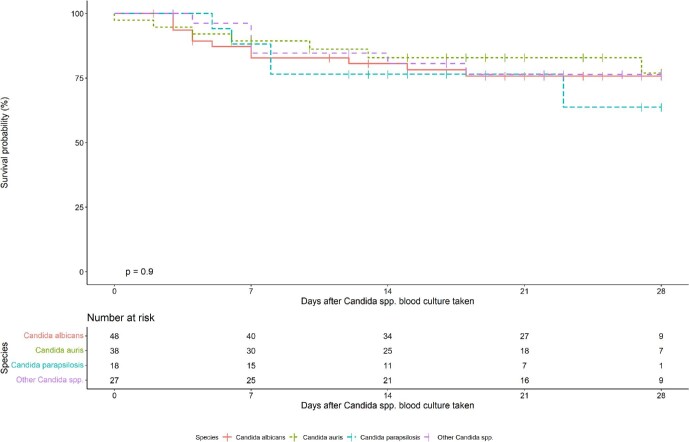
Kaplan–Meier curve for mortality from day of culture for each *Candida* species.

Death by day 28 post-enrollment was 22% (28/127). Sixty-four percent (81/127) of infants were still in the hospital on day 28 post-enrollment. The median length of follow-up from the day the *Candida* spp. culture was taken was 21 days (IQR: 11.5–27). Among infants who died, the median length of follow-up was 7 days (IQR: 4–12.25). Unadjusted mortality by species is illustrated in Fig. 5. In aunivariable Cox proportional hazards analysis, mortality was strongly associated with birthweight <1000 gr (HR: 3.83; 95% CI: 1.84–7.97) and gestational age <28 weeks (HR: 2.32; 95% CI: 1.08–4.99). There was no significant difference in mortality by species, by hospital, by country, or by study cohort (Table 2).

**Table 2.** Univariable hazard ratios from Cox proportional hazards analysis.

Mortality		*N* (%)	Univariable hazard ratio (95% CI, *P*)
**Birthweight (1000 gr**)	**≥1000 gr**	**95 (72.5)**	**-**
	**<1000 gr**	**36 (27.5)**	**3.83 (1.84**–**7.97, *P *< .001)**
**Gestational age**	**≥28 weeks**	**104 (79.4)**	**-**
	**<28 weeks**	**27 (20.6)**	**2.32 (1.08**–**4.99, *P *= .032)**
Organism	*Candida albicans*	48 (36.6)	-
	*Candida parapsilosis*	38 (29.0)	0.84 (0.32–2.16, *P *= .711)
	*Candida auris*	18 (13.7)	1.28 (0.44–3.70, *P *= .645)
	Other *Candida* spp.	27 (20.6)	0.92 (0.34–2.48, *P *= .868)
Country	Country 1	40 (30.5)	-
	Country 2	55 (42.0)	0.82 (0.35–1.94, *P *= .658)
	Country 3	13 (9.9)	0.77 (0.21–2.78, *P *= .685)
	Other*	23 (17.6)	0.80 (0.27–2.34, *P *= .681)
Hospital	Hospital 1	28 (21.4)	-
	Hospital 2	25 (19.1)	0.98 (0.30–3.22, *P *= .977)
	Hospital 3	21 (16.0)	1.10 (0.31–3.91, *P *= .883)
	Hospital 4	13 (9.9)	0.92 (0.23–3.70, *P *= .911)
	Hospital 5	11 (8.4)	1.16 (0.29–4.63, *P *= .837)
	Other*	33 (25.2)	1.11 (0.38–3.20, *P *= .851)
Enrollment cohort	Cohort 1	69 (52.7)	-
	Cohort 2	62 (47.3)	0.60 (0.29–1.27, *P *= .186)

Note: Significant covariates are bolded.

*Other should correspond to Other sites are comprised of nine hospitals, each contributing < 7 participants (range: 1–6 per site).

## Discussion

To the best of our knowledge, this is the largest multi-country cohort of neonates with *Candida* spp. bloodstream infections in the LMIC setting. Most of the neonates, included in the NeoOBS invasive candidiasis sub-study, were outside the high-risk groups for NIC as described in HIC, with 81% born after 28 weeks gestation and 73% with a birth weight >1000 gr. Although *C. albicans* was the most frequent species isolated, the species distribution varied significantly between hospitals and countries. Across the *Candida* spp. with known *in vitro* susceptibility profile, 40% were fluconazole-resistant, 18% were amphotericin B-resistant, while no resistance was reported for micafungin. (Albeit many isolates were not tested.) *Candida albicans* was reported to be highly susceptible to fluconazole and amphotericin B. In contrast, a significant proportion of *C. parapsilosis* was reported to be fluconazole-resistant, driven by high resistance rates observed in South Africa.^12^*Candida auris* was the third most common species overall, but its presence varied greatly between countries. Amphotericin B was the most common empiric antifungal used (74%), followed by fluconazole (22%) with echinocandins rarely used. Antifungal prophylaxis was infrequently used in infants who developed candidemia, even for those neonates considered at high-risk. Overall, the mortality was high (22%), and it was significantly associated with low birth weight (<1000 gr) and extreme prematurity (<28 weeks).

In the cohort of neonates with NIC described here, 37% weighed >1500 gr and 38% were ≥32 weeks of gestational age. These results are similar to other studies in LMIC,^11^,^20^ and contrast dramatically with the data from HICs, where extreme prematurity and ELBW neonates are the main high-risk groups for NIC.^21^ In 2018, e.g., the DeNIS study reported unusually high rates of NIC in a cohort of neonates in India born outside the hospital; more than a quarter of neonates with a positive blood culture (90/339, 26.5%) had *Candida* spp. isolated. Remarkably, 61.5% of those neonates weighed >1500 gr, and 73.3% were born at or after 32 weeks gestation.^3^

*Candida albicans* has been reported as the most common causative species in NIC.^10^,^22^ Increasingly, a shift in epidemiology of NIC globally has been described, with a higher rate of non-*albicans Candida* isolates in LMICs compared to HICs.^6^ The rise in non-*albicans Candida* spp. in NIC is associated with reduced susceptibility to fluconazole. This has been described in India, where MDR strains of *C. krusei* and *C. auris* have been reported^13^; and in South Africa,^23^ where surveillance has shown an increase in the number of fluconazole-resistant *C. parapsilosis*.^12^ For example, Govender et al. reported a significant shift toward *C. parapsilosis* in neonates, with 53% of all *C. parapsilosis* isolates being fluconazole-resistant and 44% and 70% cross-resistant to voriconazole and posaconazole, respectively.^12^ Other South African series report similar results.^7^,^24^,^25^ In the cohort presented here, *C. albicans* and *C. parapsilosis* accounted for 35% and 30% of the isolates, respectively. Whereas *C. albicans* remained mostly susceptible to fluconazole (91% cases), 59% of the *C. parapsilosis* isolates were fluconazole resistant, mostly from South Africa.

*Candida auris* was the third most commonly reported pathogen (14% of all the cases) in this cohort, with significant variability between countries (0%–27.5%). *Candida auris* is a rapidly emerging, multi-drug resistant, nosocomial pathogen, with high reported resistance to fluconazole and amphotericin B.^25–27^ There have been scant reports focused on invasive *C. auris* infections in neonates^28–30^; most of them are from India.^28^ Chakabrati et al. published a multi-center prospective study from 2011 to 2012; where amongst 273 neonates from three hospitals with NIC, the proportion of *C. auris* isolates was 2.2%.^13^ More recent data,^11^,^14^,^28^ together with our observations, show that *C. auris* has quickly become one of the most commonly encountered species causing NIC in LMICs.

Reported neonatal mortality attributable to NIC in LMICs varies from 20% to as high as 50%.^7^,^31^ In the cohort described here, mortality was strongly associated with low birth weight (<1000 gr) and gestational age (<28 weeks); however, we did not find a clear association with causative species or susceptibility profiles.

Antifungal prophylaxis with fluconazole targeted to neonates <1000 gr birth weight and/or <28 weeks gestation, as well as those infants with birth weight of 1000–1500 gr with additional risk factors, is a recommended strategy in neonatal units in HICs to prevent NIC.^32–36^ Based on our data, NIC in LMICs affects mostly neonates with a birth weight >1500 gr, putting in doubt the relevance of HIC neonatal fungal prophylaxis guidelines for LMICs. In addition, a high prevalence of fluconazole resistance poses an important barrier to the use of fluconazole prophylaxis in these countries. Future studies determining the clinical and health economic benefit of neonatal antifungal prophylaxis in LMIC settings are needed. Fluconazole is not the only available drug to be considered; prophylaxis with nystatin, a low cost oral antifungal, which has also been proven to have an impact on neonatal mortality and is included in the essential medicines list (EML)^37–39^ can also be considered.

Compared with the burden of bacterial sepsis, where *Klebsiella pneumoniae, Acinetobacter* spp., and *Escherichia coli* are the most commonly reported pathogens in LMICs,^40^,^41^ the burden of fungal sepsis, particularly caused by *Candida* spp. has been poorly described. For this reason, it is not possible to provide an accurate estimated burden of NIC in LMICs.^6^,^11^ Reported incidence of invasive candidiasis in pediatric intensive care units is significantly higher in LMICs (42.7 cases per 1000 admissions) compared to HICs (0.043–0.47 cases per 1000 admissions).^6^ In general, *Candida* spp. are likely to be an underreported pathogen and mostly linked to healthcare-associated infections.

This study has some key limitations. First, not all neonates with *Candida* spp. bloodstream infections presenting at these hospitals were enrolled in the study, introducing the risk of selection bias. The aims of the NeoOBS study were to describe presentation, management, and outcomes of infants with sepsis not describe incidence of these infections, and thus we were unable to quantify incidence of NIC in this cohort due to this enrollment bias. It is also possible that the seven hospitals contributing majority of the infants to this candidemia cohort had higher repeat blood culture rates than other hospitals in the NeoOBS study. Cohort 2 enrollment may have missed some infants who died prior to a positive culture result and who were unable to be consented, potentially contributing survivor bias and lower mortality for certain *Candida* species. Differences in baseline characteristics and mortality between the two enrollment cohorts were explored (Supplementary Table 1), and no significant differences were found in key risk factors, *Candida* species, or mortality, which supported combining patients from these two cohorts into one analysis. Additionally, we used hospital-reported identification and phenotypic susceptibility testing results for this analysis, which may be less accurate than MIC values and/or molecular identification techniques; moreover, the use of interpretation guidelines of antimicrobial resistance (e.g., CLSI, EUCAST, and BSAC) may vary by hospital. Finally, there were a number of isolates that did not have any susceptibility testing done or were only tested for fluconazole. Therefore, we are unable to fully evaluate resistance and appropriateness of choice of the antifungal treatment.

In conclusion, this study demonstrates that NIC is associated with significant mortality in the LMIC setting. The optimal method of prevention and treatment of this life-threatening infection requires further targeted studies. These studies should consider the epidemiological differences of NIC in LMICs compared to HICs, with an increased incidence of NIC in neonates outside the ‘high-risk’ group (<28 weeks and/or <1000 gr) and, although with significant variability between settings, higher rates of fluconazole resistances in non-*albicans Candida* species. Insights into the fungal epidemiology and susceptibility profiles are of utmost relevance in order to develop management guidelines for NIC in LMICs. Diagnostics for *Candida* species, including susceptibility testing, need to be made available and improved. Although no micafungin resistance was observed (within the few isolates tested), the role of empiric therapy with micafungin in LMICs for NIC needs to be a research priority. Micafungin has been included in the WHO-EML for children in 2021,^37^ but there are still limitations for its use in neonates, such as the lack of a defined optimal dose for those cases with meningoencephalitis.^42^,^43^ Finally, studies in LMICs are required to define which neonates might benefit from antifungal prophylaxis. As our study shows the current recommendations used in HIC targeting ‘high-risk’ neonates do not entirely apply to neonates in LMICs.

## Supplementary Material

myad010_Supplemental_Files

## References

[1] World Health Organization . Newborns: improving survival and well-being. World Health Organization. 2020. pp. 1–5.

[2] World Health Organization . Global report on the epidemiology and burden of sepsis: current evidence, identifying gaps and future directions. World Health Organization. 2020. p. 56, https://apps.who.int/iris/handle/10665/334216

[3] Jajoo M , Manchanda V, Chaurasia S et al. Alarming rates of antimicrobial resistance and fungal sepsis in outborn neonates in North India. PLoS One. 2018; 13: 1–16. 10.1371/journal.pone.0180705 PMC6023165 29953451

[4] Breiman RF , Blau DM, Mutevedzi P et al. Postmortem investigations and identification of multiple causes of child deaths: an analysis of findings from the Child Health and Mortality Prevention Surveillance (CHAMPS) network. PLoS Med. 2021; 18: 1–19. 10.1371/journal.pmed.1003814 PMC8516282 34591862

[5] Benjamin DK , Stoll BJ, Fanaroff AA et al. Neonatal candidiasis among extremely low birth weight infants: risk factors, mortality rates, and neurodevelopmental outcomes at 18 to 22 months. Pediatrics. 2006; 117: 84–92. 16396864 10.1542/peds.2004-2292

[6] Kaur H , Chakrabarti A. Strategies to reduce mortality in adult and neonatal candidemia in developing countries. J Fungi. 2017; 3: 41. 10.3390/jof3030041 PMC5715942 29371558

[7] Ballot DE , Bosman N, Nana T, Ramdin T, Cooper PA. Background changing patterns of neonatal fungal sepsis in a developing country. J Trop Pediatr. 2013; 59: 460–464. 23803724 10.1093/tropej/fmt053

[8] Morkel G , Bekker A, Marais BJ, Kirsten G, van Wyk J, Dramowski A. Bloodstream infections and antimicrobial resistance patterns in a South African neonatal intensive care unit. Paediatr Int Child Health. 2014; 34: 108–114. 24621234 10.1179/2046905513Y.0000000082

[9] Warris A , Pana Z, Oletto A, Lundin R. Antifungal drug susceptibility of candida spp. in neonatal and paediatric candidaemia: a European multi-centre retrospective study (EUROCANDY) [Abstract}. European Society of Paediatric Infectious Diaseases, Malmö, 2018 May 29.

[10] Pana ZD , Roilides E, Warris A, Groll AH, Zaoutis T. Epidemiology of invasive fungal disease in children. J Pediatric Infect Dis Soc. 2017; 6: S3–S11. 28927200 10.1093/jpids/pix046 PMC5907880

[11] Shuping L , Mpembe R, Mhlanga M et al. Epidemiology of culture-confirmed candidemia among hospitalized children in South Africa, 2012–2017. Pediatr Infect Dis J. 2021; 40: 730–737. 33872278 10.1097/INF.0000000000003151

[12] Govender NP , Patel J, Magobo RE et al. Emergence of azole-resistant *Candida parapsilosis* causing bloodstream infection: results from laboratory-based sentinel surveillance in South Africa. J Antimicrob Chemother. 2016; 71: 1994–2004. 27125552 10.1093/jac/dkw091 PMC11911919

[13] Chakrabarti A , Sood P, Rudramurthy SM et al. Characteristics, outcome and risk factors for mortality of paediatric patients with ICU-acquired candidemia in India: a multicentre prospective study. Mycoses. 2020; 63: 1149–1163. 10.1111/myc.13145 32681527

[14] Van Schalkwyk E , Mpembe RS, Thomas J et al. Epidemiologic shift in candidemia driven by *Candida auris*, South Africa, 2016–2017. Emerg Infect Dis. 2019; 25: 1698–1707. 31441749 10.3201/eid2509.190040 PMC6711229

[15] NeoOBS _ Penta Foundation . https://penta-id.org/severe-infections-and-antimicrobial-resistance/neoobs/, (February 23, 2023, date last accessed)..

[16] GARDP . Transforming the care of babies with sepsis. https://gardp.org/wp-content/uploads/2022/10/GARDP-Neonatal-sepsis-study-results-2022.pdf, (February 23, 2023, date last accessed).

[17] Harris PA , Taylor R, Thielke R, Payne J, Gonzalez N, Conde JG. Research electronic data capture (REDCap)—a metadata-driven methodology and workflow process for providing translational research informatics support. J Biomed Inform. 2009; 42: 377–381. 18929686 10.1016/j.jbi.2008.08.010 PMC2700030

[18] Harris PA , Taylor R, Minor BL et al. The REDCap consortium: building an international community of software platform partners. J Biomed Inform. 2019; 95: 103208. 31078660 10.1016/j.jbi.2019.103208 PMC7254481

[19] Russell N , Stöhr W, Plakkal N et al. Patterns of antibiotic use, pathogens and clinical outcomes in hospitalised neonates and young infants with sepsis in the NeoOBS global neonatal sepsis observational cohort study. medRxiv. https://www.medrxiv.org/content/early/2022/06/23/2022.06.20.22276674, (June 23, 2022, date last accessed). 10.1371/journal.pmed.1004179 PMC10249878 37289666

[20] Lona-Reyes JC , Gómez-Ruiz LM, Cordero-Zamora A et al. Incidencia y factores asociados a candidiasis invasiva en una unidad de cuidados intensivos neonatales de México. An Pediatría. 2022; 97: 79–86.

[21] Steinbach WJ , Roilides E, Berman D et al. Results from a prospective, international, epidemiologic study of invasive candidiasis in children and neonates. Pediatr Infect Dis J. 2012; 31: 1252–1257. 22982980 10.1097/INF.0b013e3182737427

[22] Warris A , Pana Z-D, Oletto A, Lundi R, Roilides E, EUROCANDY-Study-Group. Epidemiology and outcomes of candidaemia in neonates and children in Europe: an 10-year multinational retrospective study. 2020; 39: 114–120. 10.1097/INF.0000000000002530 PMC7208278 31725552

[23] Van Schalkwyk E , Iyaloo S, Naicker SD et al. Large outbreaks of fungal and bacterial bloodstream infections in a neonatal unit, South Africa, 2012–2016. Emerg Infect Dis. 2018; 24: 1204–1212. 29912684 10.3201/eid2407.171087 PMC6038734

[24] Pillay D , Naidoo L, Swe Swe-Han K, Mahabeer Y. Neonatal sepsis in a tertiary unit in South Africa. BMC Infect Dis. 2021; 21: 1–10. 33639864 10.1186/s12879-021-05869-3 PMC7912533

[25] Friedman DZP , Schwartz IS. Emerging fungal infections: new patients, new patterns, and new pathogens. J Fungi. 2019;5:67. 10.3390/jof5030067 PMC6787706 31330862

[26] Lockhart SR , Etienne KA, Vallabhaneni S et al. Simultaneous emergence of multidrug-resistant *Candida auris* on 3 continents confirmed by whole-genome sequencing and epidemiological analyses. Clin Infect Dis. 2017; 64: 134–140. 27988485 10.1093/cid/ciw691 PMC5215215

[27] Lockhart SR , Guarner J. Emerging and reemerging fungal infections. Semin Diagn Pathol. 2019; 36: 177–181. 31010605 10.1053/j.semdp.2019.04.010 PMC11979780

[28] Chandramati J , Sadanandan L, Kumar A, Ponthenkandath S. Neonatal *Candida auris* infection: management and prevention strategies––a single centre experience. J Paediatr Child Health. 2020; 56: 1565–1569. 32672390 10.1111/jpc.15019

[29] Berrio I , Caceres DH, Coronell RW et al. Bloodstream infections with *Candida auris* among children in Colombia: clinical characteristics and outcomes of 34 cases. J Pediatric Infect Dis Soc. 2021; 10: 151–154. 32373928 10.1093/jpids/piaa038

[30] Mesini A , Saffioti C, Mariani M et al. First case of *Candida auris* colonization in a preterm, extremely low-birth-weight newborn after vaginal delivery. J Fungi. 2021; 7: 1–4. 10.3390/jof7080649 PMC8398378 34436188

[31] Ahangarkani F , Shokohi T, Rezai MS et al. Epidemiological features of nosocomial candidaemia in neonates, infants and children: a multicentre study in Iran. Mycoses. 2020; 63: 382–394. 31985076 10.1111/myc.13053

[32] Manzoni P , Mostert M, Latino MA et al. Clinical characteristics and response to prophylactic fluconazole of preterm VLBW neonates with baseline and acquired fungal colonisation in NICU: data from a multicentre RCT. Early Hum Dev. 2012; 88: S60–S64. 22633517 10.1016/S0378-3782(12)70017-8

[33] Kaufman DA , Morris A, Gurka MJ, Kapik B, Hetherington S. Fluconazole prophylaxis in preterm infants: a multicenter case-controlled analysis of efficacy and safety. Early Hum Dev. 2014; 90: S87–S90. 24709470 10.1016/S0378-3782(14)70026-X

[34] Ericson JE , Kaufman DA, Kicklighter SD et al. Fluconazole prophylaxis for the prevention of candidiasis in premature infants: a meta-analysis using patient-level data. Clin Infect Dis. 2016; 63: 604–610. 27298330 10.1093/cid/ciw363 PMC4981761

[35] Swanson JR , Vergales J, Kaufman DA, Sinkin RA. Cost analysis of fluconazole prophylaxis for prevention of neonatal invasive candidiasis. Pediatr Infect Dis J. 2016; 35: 519–523. 26835970 10.1097/INF.0000000000001068

[36] Hope WW , Castagnola E, Groll AH et al. ESCMID * guideline for the diagnosis and management of Candida diseases 2012 : prevention and management of invasive infections in neonates and children caused by *Candida* spp. Clin Microbiol Infect. 2012; 18: 38–52. 23137136 10.1111/1469-0691.12040

[37] World Health Organization . World Health Organization. Model List of Essential Medicines–22nd List, 2021. 2021. https://www.who.int/publications/i/item/WHO-MHP-HPS-EML-2021.02, (February 23, 2023, date last accessed).

[38] Kaufman DA . “Getting to Zero”: preventing invasive *Candida* infections and eliminating infection-related mortality and morbidity in extremely preterm infants. Early Hum Dev. 2012; 88: S45–S49. 22633513 10.1016/S0378-3782(12)70014-2

[39] Rundjan L , Wahyuningsih R, Oeswadi CA, Marsogi M, Purnamasari A. Oral nystatin prophylaxis to prevent systemic fungal infection in very low birth weight preterm infants : a randomized controlled trial. BMC Pediatr. 2020; 20: 170–179. 32303210 10.1186/s12887-020-02074-0 PMC7164192

[40] Downie L , Armiento R, Subhi R, Kelly J, Clifford V, Duke T. Community-acquired neonatal and infant sepsis in developing countries: efficacy of WHO's currently recommended antibiotics––Systematic review and meta-analysis. Arch Dis Child. 2013; 98: 146–154. 23142784 10.1136/archdischild-2012-302033

[41] Russell N , Stoehr W, Plakkal N, Cook A. Analysis from the NeoOBS global neonatal sepsis prospective observational cohort study across 19 hospitals in 11 countries; clinical presentation, treatment, mortality outcomes and develpment of the NeoSEP sepsis severity score. Available at SSRN, https://ssrn.com/abstract=3864901, (June 21, 2021, date last accessed).

[42] Taormina G , Gopinath R, Moore J et al. A regulatory review approach for evaluation of micafungin for treatment of neonatal candidiasis. Clin Infect Dis. 2021; 73: 2335–2340. 33458754 10.1093/cid/ciab025

[43] Leroux S , Jacqz-Aigrain E, Elie V et al. Pharmacokinetics and safety of fluconazole and micafungin in neonates with systemic candidiasis: a randomized, open-label clinical trial. Br J Clin Pharmacol. 2018; 84: 1989–1999. 29744900 10.1111/bcp.13628 PMC6089805

