# Supplementary material for: Neonatal invasive candidiasis in low- and middle-income countries: Data from the NeoOBS study
Source: Med Mycol. 2023 Mar 6;61(3):myad010. doi: 10.1093/mmy/myad010 (PMC10026246; doi:10.1093/mmy/myad010)
Supplement: myad010_Supplemental_Files [file myad010_supplemental_files.zip › mm-2022-0139-File009.docx]

**Supplemental Figure 1**

a)


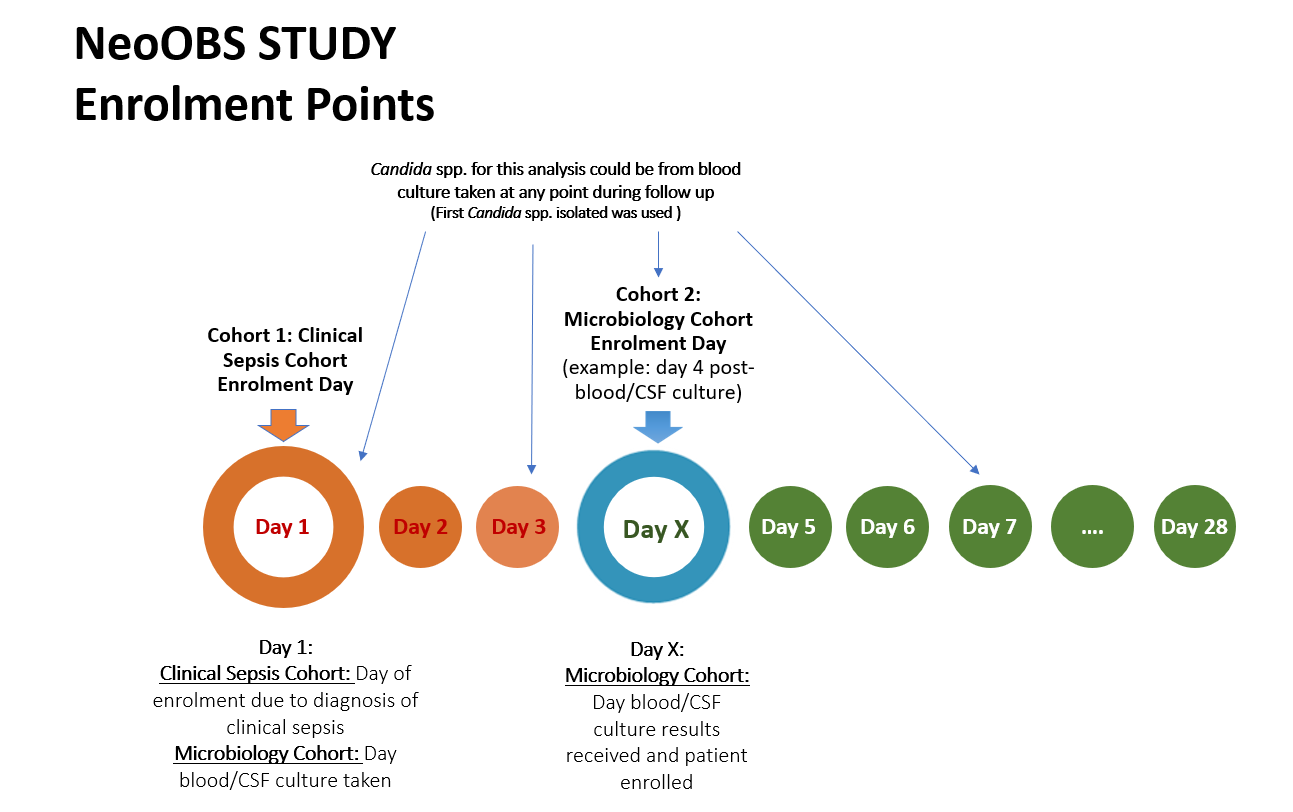


b)


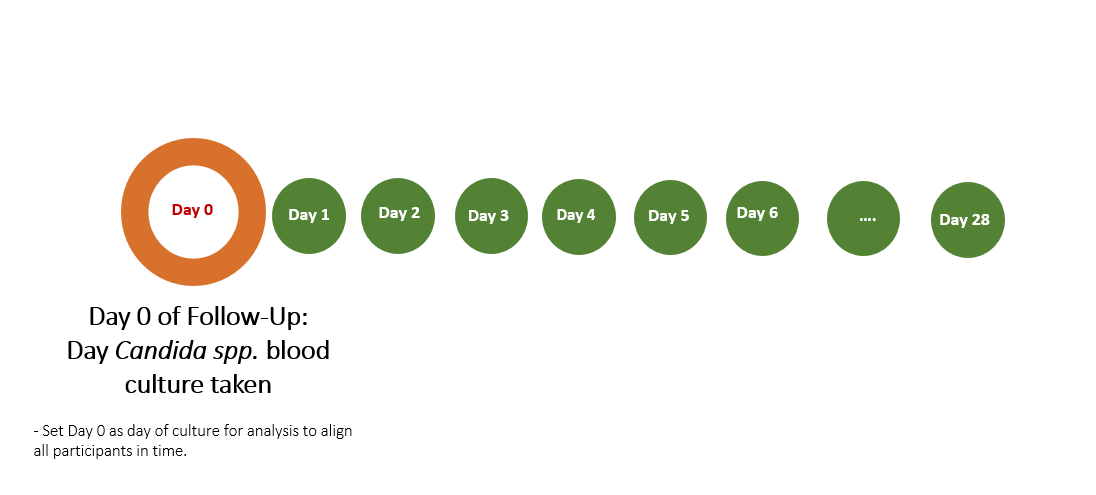


Supplemental Figure 1. Schematic of the study population indicating: a) the two enrolment cohorts for the overall NeoOBS study population and b) the *Candida spp.* sub-study timeline. Infants from either enrolment cohort with a *Candida* spp. isolated from blood culture at any point during follow up were included in the analysis and standardised with day the culture was taken as day 0 for this analysis.
