# Supplementary material for: Neonatal invasive candidiasis in low- and middle-income countries: Data from the NeoOBS study
Source: Med Mycol. 2023 Mar 6;61(3):myad010. doi: 10.1093/mmy/myad010 (PMC10026246; doi:10.1093/mmy/myad010)
Supplement: myad010_Supplemental_Files [file myad010_supplemental_files.zip › mm-2022-0139-File010.docx]

**Supplemental Table 1. Detailed inclusion criteria for the overall NeoOBS study (cohort 1 and cohort 2)**

|  | **Cohort 1** | **Cohort 2** |
| --- | --- | --- |
| **Inclusion criteria** | - In-patient in the hospital of this institution (neonatal unit, paediatric ward, or emergency department) - Age <60 days (postnatal age) - Informed consent from parent/guardian - Parent provided contact information for 28-day follow-up - Clinical suspicion of a new episode of sepsis (defined below*) together with planned treatment with IV antibiotics | - In-patient in the hospital of this institution (neonatal unit, paediatric ward, or emergency department) - Age <60 days (postnatal age) - Informed consent from parent/guardian - Parent provided contact information for 28-day follow-up - One of the following microbiology findings:   o New episode of infection in which a Candida species is isolated from blood culture  OR  o New episode of infection in which a carbapenem-resistant organism (CRO) is isolated from blood culture  OR  o New episode of confirmed bacterial meningitis – defined as (A) or (B) below  (A) Isolation of a significant bacterial pathogen from cerebrospinal fluid (CSF)  (B) Isolation of a significant bacterial pathogen from blood cultures AND CSF white cells ≥20 cells/mm3 (for babies 0-28 days of age) or CSF white cells ≥ 10 cells/mm3 (for babies 29-60 days of age) |
| **Exclusion Criteria** | - Previously enrolled in this study - Enrolment in any interventional trial - A serious, non-infective co-morbidity (other than prematurity) that is anticipated to cause death within 72 hours | |
| ***Definition of clinical sepsis** | Baby must have at least two clinical/laboratory signs of which at least one must be a clinical sign. Second criteria may be either clinical or laboratory sign.  Clinical Criteria   - Capillary refill time (CRT) >3 sec or mottled skin or other evidence of shock - Multiple or severe skin pustules - Petechial rash - Pus from umbilical stump - Severe chest in-drawing or increased oxygen requirement or need for respiratory support - Grunting - Apnoea - Cyanosis - Abnormal heart rate (>180/min or <100/min) - Abnormal temperature (>37.5°C or <36.5°C) or temperature instability (i.e. wide variations) - Irritability - Convulsions - Abnormal posturing - Hypotonia/floppiness - Lethargy or drowsiness - Bulging fontanelle - No movement or movement only when stimulated - Difficulty feeding or feeding intolerance - Abdominal distension   Laboratory Criteria   - White blood cells (WBC) count <4x109 cells/L or >20x109 cells/L - Absolute neutrophil count <1.5x109 cells/L - Immature-to-total (ITT) polymorph ratio >0.2 - C-reactive protein (CRP) >10mg/L or >1 mg/dL - Acidosis: Base excess < -10mmol/L OR blood lactate >2 mmol/L | |
