# Supplementary material for: Neonatal invasive candidiasis in low- and middle-income countries: Data from the NeoOBS study
Source: Med Mycol. 2023 Mar 6;61(3):myad010. doi: 10.1093/mmy/myad010 (PMC10026246; doi:10.1093/mmy/myad010)
Supplement: myad010_Supplemental_Files [file myad010_supplemental_files.zip › mm-2022-0139-File011.docx]

**Supplemental table 2.** Comparison of characteristics by overall enrolment cohort.

|  | **Overall (n=127)** | **Cohort 1 (n=67)** | **Cohort 2 (n=60)** | **p** |
| --- | --- | --- | --- | --- |
| **Sex = Female** (%) | 59 (47) | 27 (40) | 32 (54) | 0.196 |
| **Birth weight (g)** (median [IQR]) | 1270.0  [990.0, 1692.5] | 1195.0  [905.0, 1547.5] | 1420.0  [1023.8, 1900.0] | 0.041 |
| **Gestational age (weeks**) (median [IQR]) | 30.0  [28.0, 34.0] | 30.0  [28.0, 32.0] | 30.0  [28.0, 35.3] | 0.301 |
| **Postnatal age at Candida spp. culture (days**) (median [IQR]) | 16.0  [10.5, 22.0] | 17.0  [12.5, 25.0] | 14.5  [9.8, 20.0] | 0.026 |
| **Birth status = Hospitalised since birth** (%) | 114 (90) | 65 (97) | 49 (82) | 0.011 |
| **Organism(n=128)** (%) |  |  |  | 0.108 |
| *Candida albicans* | 45 (35) | 29 (43) | 16 (27) |  |
| *Candida parapsilosis* | 38 (30) | 17 (25) | 21 (35) |  |
| *Candida auris* | 18 (14) | 11 (16) | 7 (12) |  |
| Other *Candida* spp. | 27 (21) | 10 (15) | 17 (28) |  |
| **Country** (%) |  |  |  | 0.157 |
| India | 40 (32) | 19 (28) | 21 (35) |  |
| South Africa | 55 (43) | 35 (52) | 20 (33) |  |
| Vietnam | 13 (10) | 6 (9) | 7 (12) |  |
| Other* | 19 (15) | 7 (10) | 12 (20) |  |
| **Hospital** (%) |  |  |  | 0.624 |
| Hospital 1 | 28 (22) | 16 (24) | 12 (20) |  |
| Hospital 2 | 25 (20) | 13 (19) | 12 (20) |  |
| Hospital 3 | 21 (17) | 14 (21) | 7 (12) |  |
| Hospital 4 | 13 (10) | 6 (9) | 7 (12) |  |
| Hospital 5 | 11 (9) | 4 (6) | 7 (12) |  |
| Other* | 29 (23) | 14 (21) | 15 (25) |  |
| **Mortality = Died** (%) | 28 (22) | 16 (24) | 12 (20) | 0.755 |

*^a^*Other *Candida* spp. includes*: Candida famata (n=1), Candida glabrata (n=6), Candida metapsilosis (n=1), Candida pelliculosa (n=4), Candida rugosa (n=1), undefined Candida spp. (n=10), Candida tropicalis (n=4)*

*^b^Other countries is comprised of 5 countries, each contributing <8 participants (range: 1-7 per country).*

*^c^Other sites is comprised of 9 hospitals, each contributing <7 participants (range: 1-6 per site)*
