# Supplementary material for: Neonatal invasive candidiasis in low- and middle-income countries: Data from the NeoOBS study
Source: Med Mycol. 2023 Mar 6;61(3):myad010. doi: 10.1093/mmy/myad010 (PMC10026246; doi:10.1093/mmy/myad010)
Supplement: myad010_Supplemental_Files [file myad010_supplemental_files.zip › mm-2022-0139-File012.docx]

**Supplemental table 3**. Comparison of *Candida* species by country and hospital.

|  |  | *Candida albicans* | *Candida parapsilosis* | *Candida auris* | Other *Candida* spp. | Total | p |
| --- | --- | --- | --- | --- | --- | --- | --- |
| Country | India | 10 | 9 | 11 | 10 | 40 | <0.0001 |
|  | South Africa | 23 | 23 | 7 | 2 | 55 |  |
|  | Vietnam | 8 | 3 | 0 | 2 | 13 |  |
|  | Other^a^ | 4 | 3 | 0 | 13 | 23 |  |
| Hospital | Hospital 1 | 12 | 10 | 5 | 1 | 28 | <0.0001 |
|  | Hospital 2 | 3 | 5 | 11 | 6 | 25 |  |
|  | Hospital 3 | 7 | 12 | 2 | 0 | 21 |  |
|  | Hospital 4 | 8 | 3 | 0 | 2 | 13 |  |
|  | Hospital 5 | 4 | 3 | 0 | 4 | 1 |  |
|  | Other^b^ | 11 | 5 | 0 | 14 | 33 |  |

*^a^Other countries is comprised of 5 countries, each contributing <8 participants (range: 1-7 per country).*

*^b^Other sites is comprised of 9 hospitals, each contributing <7 participants (range: 1-6 per site)*
